# Freeform thin-film lithium niobate mode converter for photon-pair generation

**DOI:** 10.1515/nanoph-2024-0515

**Published:** 2025-02-06

**Authors:** Changhyun Kim, Munseong Bae, Minho Choi, Sangbin Lee, Myunghoo Lee, Chihyeon Kim, Hojoong Jung, Haejun Chung, Hyounghan Kwon

**Affiliations:** Center for Quantum Technology, 58975 Korea Institute of Science and Technology (KIST), Seoul 02792, South Korea; Department of Electronic Engineering, Hanyang University, Seoul, 04763, South Korea; Department of Artificial Intelligence Semiconductor Engineering, Hanyang University, Seoul, 04763, South Korea; Department of Electrical and Computer Engineering, University of Washington, Seattle, WA, 98195, USA; Quantum Technology Institute, Korea Research Institute of Standards and Science (KRISS), Daejeon, 34113, South Korea; Department of Electronic Engineering, Department of Artificial Intelligence, and Department of Artificial Intelligence Semiconductor Engineering, Hanyang University, Seoul, 04763, South Korea; Division of Quantum Information, KIST School, Korea University of Science and Technology, Seoul 02792, South Korea

**Keywords:** lithium niobate, mode converter, inverse design, topology optimization, spontaneous parametric down conversion

## Abstract

Thin-film lithium niobate (TFLN) has emerged as a promising platform for integrated photonics due to its exceptional material properties. The application of freeform topology optimization to TFLN devices enables the realization of compact designs with complex functionalities and high efficiency. However, the stringent fabrication constraints of TFLN present significant challenges for optimization, particularly in nonlinear photonic devices. In this work, we propose an inverse design methodology that successfully addresses these challenges and demonstrates the development of an efficient freeform TFLN mode converter. The numerically optimized mode converter achieves a transmission efficiency of 67.60 % and a mode purity of 84.58 %. Experimental validation through nonlinear processes, including second harmonic generation and spontaneous parametric down-conversion, shows that the fabricated devices improve the efficiency of these processes by factors of two and three, respectively, compared to devices without freeform designs. The proposed inverse design framework provides a powerful tool for advancing the development of TFLN-based devices, with broad applicability to nonlinear and quantum photonics.

## 1 Introduction

Integrated photonics is driving advances in modern optical technology by incorporating multiple optical components onto a single chip. This shift toward photonic integration offers significant benefits, including reduced form factor and strong light–matter interaction [1]. Thanks to its unprecedented functionality and scalability, the integrated photonics plays a key role in the development of nonlinear and quantum optics devices [1], [2], [3], [4]. Among the diverse material platforms of integrated photonics, thin-film lithium niobate (TFLN) on insulator has emerged as a transformative material [5], [6]. TFLN provides a unique combination of high electro-optic coefficients, a broad transparency window, low optical losses, and second-order nonlinearity, making it an ideal candidate for next-generation active, nonlinear, or quantum photonic devices [7], [8], [9]. In addition, its compatibility with existing semiconductor fabrication processes facilitates the integration of TFLN-based devices with other materials and components, paving the way for more sophisticated and scalable photonic systems [10], [11]. As a result of these exceptional properties, various TFLN on-chip devices have been demonstrated, such as electro-optic modulators [12], [13], [14], frequency combs [15], [16], squeezed light sources [17], [18], optical parametric processes [18], [19], and entangled photon pair generation [20], [21].

The design of TFLN devices necessitates meticulous consideration of fabrication constraints, including etching depth, slanted sidewalls, and minimum size constraints, to ensure robustness in the fabrication process. For example, failure to satisfy minimum gap size constraints can result in the formation of multiple micro trenches during fabrication, leading to significant deviations between the intended design and the fabricated device. Similarly, violating the minimum feature size constraint, determined governed by the thickness of the etching masks, can cause small features to anneal during fabrication process. Nonlinear photonic devices tend to possess even severe constraints because they often necessitate the thick etching depth [15], [22], [23]. As a result, designs of the TFLN nonlinear and quantum photonic devices mostly rely on intuition-based approaches through a sweep of small sets of design parameters like length, width, and gap size [23], [24], [25].

Inverse design methods [26], [27], [28] facilitate exploration of non-intuitive photonic design spaces, enabling the development of high-efficiency, multifunctional devices [29], [30], [31], [32], [33], [34], [35], [36], [37], [38]. Specifically, topology optimization with adjoint variable method, also known as adjoint optimization, leverages the Born approximation and Lorentz reciprocity [39], which accelerates the computation of partial derivatives of predefined objectives with respect to changes in design parameter (e.g., material density) across the entire design space. Recent advancements in the inverse design of linear TFLN devices, considering slanted sidewalls [40], have made it possible to design freeform TFLN devices while adhering to precise fabrication constraints. However, enforcing fabrication constraints in inverse design methods typically relies on density filters [41], [42], [43]. While these filters are scaled to match minimum size constraints, their use often excessively blurs the filtered design fields, thereby limiting design freedom [44]. This presents a significant challenge in achieving compact designs under the severe fabrication constraints of thick nonlinear TFLN-integrated photonic elements. In addition, shape-optimized TFLN mode converters enable efficient spontaneous parametric down conversion (SPDC) for diverse modal phase matching conditions [21]. However, the proposed method in ref. [21], impose limitations on the design space and assume perpendicular sidewalls during the optimization process. These assumptions result in larger footprints compared to freeform structures and lead to suboptimal device performance.

In this work, we present an inverse-designed freeform TFLN mode converter. By employing adjoint optimization with a precise mapping function, we discover the optimal geometry of the freeform waveguide for targeted mode conversion. This approach ensures consistency between the optimized design and the fabricated device, despite severe fabrication constraints inherent in thick nonlinear TFLN-integrated photonic elements with type-0 phase matching condition. The optimized mode converter, with a compact footprint of 5.6 × 7.6 μm^2^, achieves a numerical transmission efficiency of 67.60 % and a mode purity of 84.58 %. The strict design constraints were preserved throughout the fabrication process. Furthermore, second harmonic generation (SHG) and SPDC experiments using the fabricated device demonstrated a 2- to 3-fold increase in efficiency compared to devices without our freeform mode converter.

## 2 Method and simulation results

### 2.1 Inverse design of freeform TFLN mode converter

We employ the inverse design method in three-dimensional (3D) finite-difference time-domain (FDTD) simulations with the grid size of 20 nm to design the freeform TFLN mode converter that satisfies the constraints of the TFLN waveguide. The figure of merit (FoM) is defined as the denominator of the mode purity at the output waveguide:

(1) $$\chi =\frac{|\underset{S}{\iint }E_{\text{sim}}\cdot E_{TE_{20}}^{*}\;dS{|}^{2}}{\underset{S}{\iint }|E_{\text{sim}}{|}^{2}\;dS\underset{S}{\iint }|E_{TE_{20}}{|}^{2}\;dS},$$

where *χ* is mode purity, *E*_sim_ is the simulated electric field profile at the output waveguide, and $E_{{TE}_{20}}$ is the electric field profile of an ideal TE_20_ mode.

**Figure 1:**
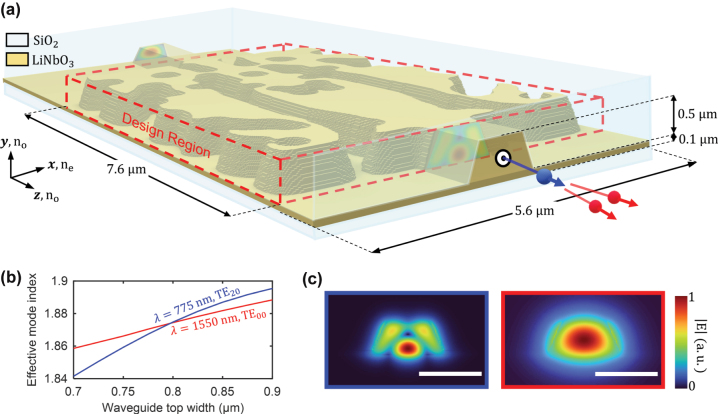
Freeform mode converter on thin-film lithium niobate (TFLN) designed to generate a high-order target mode for high-efficiency SPDC. (a) Schematic of the freeform mode converter on a 100 nm thick LN substrate, which converts the TE_00_ mode to the target mode (TE_20_) at a wavelength of 775 nm (*λ*). The total thickness of LN film is 600 nm, including the etched layer and substrate. (b) Effective mode index curves for the target mode (TE_20_, 775 nm, blue curve) and the down-converted mode (TE_00_, 1,550 nm, red curve). The effective mode indices match at a waveguide top width of 798 nm. (c) Normalized electric field amplitude profiles of the TE_20_ mode at 775 nm (left) and the TE_00_ mode at 1,550 nm (right). The scale bar represents 1 μm.

Figure 1(a) illustrates the schematic of the photon pair generation process using an inverse-designed freeform mode converter within a 5.6 × 0.5 × 7.6 μm^3^ design region on a 100 nm thick *X*-cut TFLN substrate. The converter transforms the input TE_00_ mode into the desired high-order mode at a wavelength of 775 nm. The converted mode then propagates through the phase-matching waveguide, generating photon pairs in the telecom band. To achieve type-0 phase matching, the high-order target mode and the top width of the phase-matching waveguide are set as TE_20_ and 798 nm, respectively [21]. Figure 1(b) shows the calculated effective mode index curves for the TE_20_ mode at 775 nm and the TE_00_ mode at 1,550 nm as a function of waveguide top width. Figure 1(c) presents the electric field profiles of the target and down-converted modes in the vertical cross-section of the phase-matching waveguide. To reduce the computational complexity of the TFLN mode converter design, mirror symmetry is imposed along the *y*–*z* plane (with *x* as the center), leveraging the inherent symmetry of the TE_00_ and TE_20_ modes at 775 nm.

### 2.2 Design framework for freeform TFLN devices

**Figure 2:**
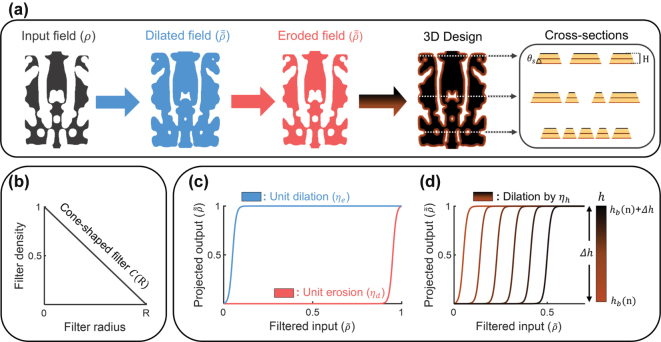
Overview of our inverse design methodology to implement the fabrication constraints for general TFLN devices (a) through multiple dilation and erosion processes, the input design field evolves into the top layer of the design, ensuring a minimum feature size and a minimum gap size between local features. The top layer then undergoes additional height-dependent dilation processes to achieve slanted sidewalls. Each dilated layer is stacked into the 3D structure with a satisfying etching depth of *H* (500 nm) and side angle of *θ*_*s*_ (67°). (b) Standard cone-shaped filter with radius *R* for each process of the mapping function. (c) The hyperbolic tangent projection functions with threshold values *η*_*d*_ (0.05) and *η*_*e*_ (0.95) for unit dilation (blue curve) and erosion (red curve), respectively. (d) The projection function for the dilation by height-dependent threshold function (*η*_*h*_) in the *n*_th_ subsection of the TFLN design. For each section, the top layer is dilated with the linearly shifted threshold value from *h* = *h*_*b*_(*n*) + Δ*h* (0.5) to *h* = (*h*_*b*_(*n*)) (0.05).

Our inverse design method combines a gradient-based topology optimization solver [42] with an external parameter update algorithm that leverages adjoint gradient analysis through a mapping function specifically designed to address the fabrication constraints of the nonlinear TFLN platform. Each iteration of the inverse design process begins with the forward computation of the mapping function, which converts the two-dimensional (2D) input design field into a three-dimensional (3D) structure that adheres to the defined geometric constraints, as illustrated in Figure 2(a). Following the computation of the mapping function, the optimization solver evaluates the predefined FoM and calculates adjoint gradients across the entire design region using two FDTD simulations [45]. The 3D adjoint gradient profile is then backpropagated through the mapping function, yielding a gradient map that aligns with the dimensions of the input design field. Finally, the gradient ascent algorithm utilizes this gradient map to update the input design field for the subsequent iteration.

The TFLN applications require additional geometric constraints, including a minimum gap size (MGS) between neighboring features and a minimum feature size (MFS) within the design region. In this work, we define the lower bound of the MGS for the top layer (MGS_top_) as 423 nm to ensure slanted sidewalls at an angle of 67° with an etching depth of 500 nm. The lower bound of the MFS (MFS_top_) is set at 100 nm, conservatively determined by the e-beam writing and dry etching process. However, defined MGS_top_ exceeds half of the target wavelength, limiting sub-wavelength effects during the optimization process and potentially leading to a bulky design with limited performance.

To overcome these limitations, we utilize the mapping function comprising multiple dilation and erosion processes [44]. This mapping function generates the extended input design field with strict constraints while avoiding local eliminations, thereby preserving greater design flexibility. During the backpropagation process, the mapping function facilitates the transformation of the 3D adjoint gradient profile into a gradient map that incorporates the imposed constraints, ensuring that the input design field remains consistent with the design objectives.

Figure 2(a) illustrates the three primary steps of the mapping function employed to maximize design freedom while ensuring compliance with the fabrication constraints in the inverse design of TFLN devices. In the first step, the 2D input design field is extended into the bottom layer of the design using dilation processes. These processes continue until the MFS of the dilated design field reaches MFS_bot_ (calculated as MFS_top_ + MGS_top_). Subsequently, the dilated field evolves into the top layer of the design through the unit erosion processes until MFS_top_ and MGS_top_ are secured without any elimination of local features. Finally, the produced top layer of the design is extended by height-dependent dilations and stacked into the 3D design with the desired etching depth (*H* = 500 nm) and side angle (*θ*_*s*_ = 67°).

Each step of the mapping function consists of two sequential sub-processes, as shown in Figure 2(b)–(d), which are performed using identical parameters (e.g., filter radius and threshold value). Each sub-process begins with the convolution between the input design field with the standard cone-shaped filter as follows:

(2) $$\tilde{\rho }=\rho *C\left(R\right),R\in \left\{R_{u},R_{s}\right\},$$

where *ρ* is the input design field, $\tilde{\rho }$ is the filtered design field, and *C*(*R*) is a standard cone-shaped filter with minimized radius *R* in Figure 2(b). To implement filter- and projection-based erosion and dilation, at least two points are required along the shortest path, along with the maximum and minimum values of the filtered fields. These values are linearly interpolated, with at least three odd-numbered points, including the midpoint, necessary to perform sequential erosions and dilations under the same filter radius. The filter radius determines the minimum number of points, expressed as a natural number, *N*, in units of the grid size (20 nm). The corresponding filter radius equals 20 nm multiplied by (*N* + 1). Accordingly, the minimum *N* required to perform unit erosions and dilations is 3, yielding a filter radius (*R*_*u*_) of 80 nm. Similarly, the minimum *N* required to execute height-dependent dilations is 2, with a corresponding filter radius (*R*_*s*_) of 60 nm. The minimized filter radius enables consistent dilations and erosions during the main steps of our mapping function for precise fabrication constraints, preventing excessive blurring over the filtered design field.

Figure 2(c) and (d) depict the hyperbolic tangent projection functions for the filtered input design field, defined as follows [46]:

(3) $$\begin{array}{c} \tilde{\tilde{\rho }}=\frac{\tanh\left(\beta \cdot \eta \right)+\tanh\left(\beta \cdot \left(\tilde{\rho }-\eta \right)\right)}{\tanh\left(\beta \cdot \eta \right)+\tanh\left(\beta \cdot \left(1-\eta \right)\right)},\\ \beta \in \left[0,\infty \right),\eta \in \left\{\eta_{d},\eta_{e},\eta_{h}\right\}, \end{array}$$

where $\tilde{\tilde{\rho }}$ is the projected design field, *β* is the sharpness of projection, and *η* is threshold value. Specifically, *η*_*d*_ and *η*_*e*_ correspond to the threshold values for unit dilation (0.05) and erosion processes (0.95), respectively, as shown in Figure 2(c). Both polarized threshold values minimize the filter radius *R*_*u*_.

Figure 2(d) indicates linearly shifted projection functions by *η*_*h*_ as follows:

(4) $$\begin{array}{c} \eta_{h}=\frac{h-h_{b}\left(n\right)}{\Delta h}\left(0.5\eta_{d}-\eta_{d}^{2}\right),h\in \left[0,H\right],\\ h_{b}\left(n\right)=H-n\Delta h,n\in N,h_{b}\left(n\right)\in \left[0,h_{b}\left(1\right)\right], \end{array}$$

where *η*_*h*_ is the height-dependent threshold function from the top (*h*_*b*_(*n*) − Δ*h*) to bottom (*h*_*b*_(*n*)) of *n*_th_ local subsection with the size of Δ*h* (*λ*/8) for the height *h* indicating a distance from the top (*H* = 500 nm) to bottom(0) of the 3D design space. The 3D design generated by the mapping function features a fully connected geometry, free from any vanishing layers along the height axis, thereby preventing the formation of micro-trenches in the fabricated devices. The constant *β* can be used to control the overall mapping process, enabling faster or slower geometric convergence.

### 2.3 Design and simulation results

**Figure 3:**
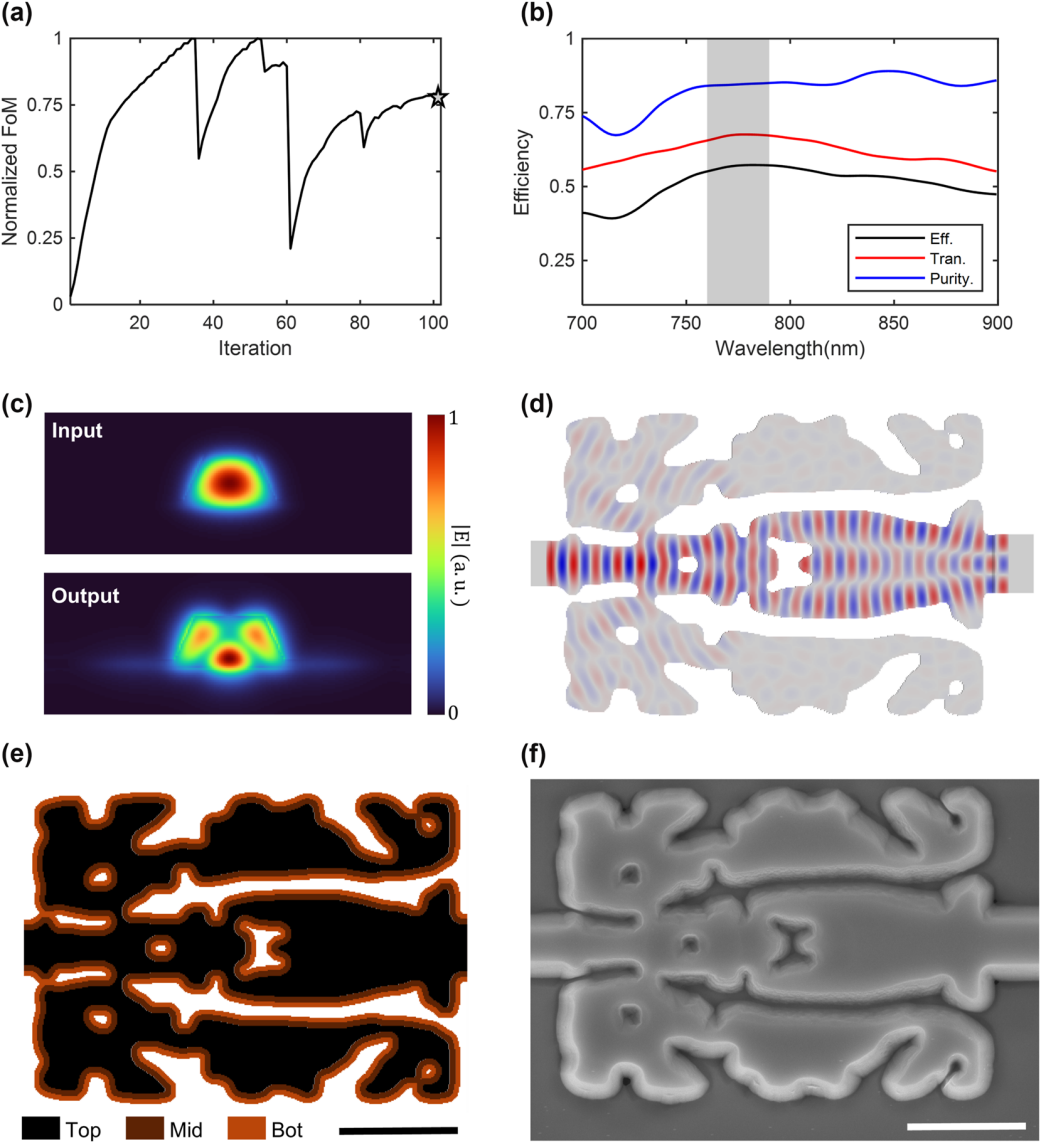
Inverse design and simulation results of the optimized freeform mode converter. (a) Inverse design iterations and the corresponding figure of merit (FoM). The marker corresponds to the FoM of the final design. (b) Simulated efficiencies of the freeform mode converter. The red and blue curves indicate the transmission and the mode purity of the TE_20_ mode, respectively. The conversion efficiency, shown as the black curve, is calculated as the product of these two curves. The gray-shaded region encompasses the bandwidth of the tunable laser. (c) Normalized electric field amplitude profiles of the vertical cross-section at the input (top) and the output (bottom) of the mode converter. (d) A middle layer (250 nm from the substrate) of the design and simulated field (**Re**[*E*_*x*_]) propagation of the freeform mode converter with a 5.6 × 7.6 μm^2^ footprint. The gray and white regions represent the LN and clad regions, respectively. (e) Top view of the optimized mode converter design. (f) Scanning electron microscopy (SEM) image of the fabricated mode converter. The SEM image is taken before the silicon dioxide PECVD process. The scale bars in (e) and (f) represent 2 μm.

Figure 3 presents the optimization results for our freeform mode converter with a 5.6 × 7.6 μm^2^ footprint, connected to input and output waveguides with top widths of 600 nm and 798 nm, respectively. Figure 3(a) shows the normalized FoM across iterations. The curve in Figure 3(a) is normalized by the maximum FoM achieved during the iterations. The process begins with a homogeneous initial geometry at a *β* = 1, which doubles when the FoM approaches saturation. In the early iterations, the adjoint gradients dominate, allowing the design to explore a broader solution space with minimal influence from the fabrication constraints imposed by *β*. Once the FoM reaches a local optimum, the impact of fabrication constraints becomes more significant, transitioning the grayscale design into a binarized final geometry with *X*-cut LN and SiO_2_ cladding. The sharp decline in the FoM curve seen in Figure 3(a) results from relatively large geometric updates followed by the changes in *β*, which could be mitigated by implementing more gradual updates to *β*.

The FDTD simulation was conducted for the optimized freeform mode converter, with an output waveguide extended to a length of 40 μm to eliminate the non-propagating modes present at the entrance of the output waveguide. Figure 3(b) indicates simulated transmission (red curve), mode purity (blue curve), and conversion efficiency (black curve) of the optimized mode converter. To be specific, the transmission is computed by the transmitted flux divided by the input flux, the conversion efficiency is calculated by multiplying the transmission and mode purity. At 775 nm, the optimized mode converter demonstrates a numerical transmission efficiency of 67.60 % and a mode purity of 84.58 %. The transmission efficiency and mode purity exhibit deviations of less than 2.5 % across the 760–790 nm wavelength range, which aligns with the bandwidth of the tunable laser for the post-fabrication measurements. These results suggest that the optimized mode converter demonstrates robustness to fabrication errors. Notably, this work represents a significant advancement compared to our previous work on the shape-optimized mode converter [21]. Specifically, the proposed freeform mode converter achieves superior mode conversion efficiency compared to the 15 μm long shape-optimized device while maintaining a considerably smaller footprint. Furthermore, this study introduces a generalized inverse design methodology for freeform photonic devices, effectively addressing the stringent fabrication constraints associated with nonlinear TFLN devices throughout the optimization process.

Figure 3(c) and (d) depict the field profiles of the optimized mode converter at the vertical and horizontal cross-sections, obtained from FDTD simulations at a wavelength of 775 nm. The normalized electric field amplitude profiles at the output waveguide exhibit a clear correspondence with the target TE_20_ mode without non-propagating modes, as shown in Figure 1(c). In further detail, the output mode in Figure 3(c) is predominantly contributed by the target TE_20_ mode, accounting for 84.58 % of the total energy. Smaller contributions are observed from the TE_01_ mode (5.03 %), the TM_30_ mode (6.9 %), and other propagating modes (3.48 %). Figure 3(d) presents the middle layer (*y* = 250 nm from the substrate) of the optimized design, overlaid with the simulated field profile. At the input waveguide, the fundamental mode (TE_00_) is clearly visible, while over the course of propagation, the fundamental mode is converted into higher-order modes.

The top-view of the optimized mode converter in Figure 3(e) and fabricated freeform TFLN mode converter in Figure 3(f) clearly reveal the compatibility of the inverse design device with the fabrication of the TFLN photonic devices. We use conventional nanofabrication techniques, including e-beam lithography, dry etching, and deposition of a silicon oxide layer for cladding [21]. Using e-beam lithography (JEOL, JBX9300FS) and hydrogen silsesquioxane (HSQ) e-beam resist, we define the patterns of inverse-designed devices and waveguides. The patterns are transferred into the TFLN layer using the dry etching technique, followed by a cleaning process. Finally, a 2 μm silicon oxide layer is deposited using plasma-enhanced chemical vapor deposition.

## 3 Experimental results

### 3.1 Second harmonic generation measurement

**Figure 4:**
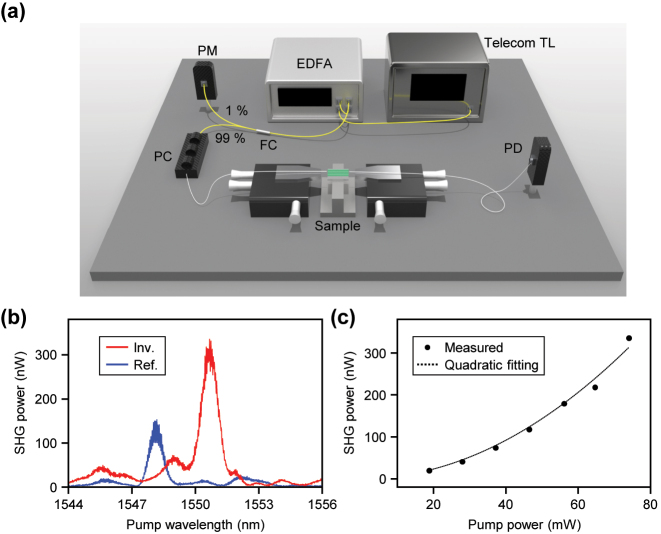
Experimental results of measuring SHG. (a) Schematic illustration of the experimental setup for SHG measurement. TL, tunable laser; EDFA, erbium-doped fiber amplifier; FC, fiber coupler; PC, polarization controller; PD, photodetector. (b) Measured SHG power with respect to the pump wavelength. The red line indicates the measured SHG spectrum of our inversely designed waveguide, and the blue line is a reference waveguide that our inversely designed mode converter is excluded. (c) Measured SHG power with respect to pump power. The dotted line indicates a quadratic fitting of the measured power.

SHG experiments are conducted using the fabricated freeform mode converters and 7.3 mm long phase-matched waveguides. The detailed experimental setup is depicted in Figure 4(a). Light from a tunable telecom laser (Santec TSL-550) is first amplified using an erbium-doped fiber amplifier (EDFA, Pritel). The amplified light is then split by a 99:1 fiber coupler, with 1 % directed to a power monitor and remaining 99 % is delivered to the sample. Prior to entering the sample, a fiber polarization controller is used to ensure the desired TE polarization state on-chip. The light is coupled into the sample via a lensed fiber. On-chip, SHG occurs in a straight waveguide configured for the TE_20_ mode. The SHG light generated in the TE_20_ mode is then converted to the TE_00_ fundamental mode using our freeform mode converter. This mode conversion expects to increase the efficiency of light collection by the output lensed fiber. Finally, the collected second harmonic light is directed to a photodetector for measurement.

The graph in Figure 4(b) presents the measured SHG results using the inverse-design mode converter device shown in Figure 3(f). For comparison, the SHG results for a reference waveguide with the same specifications but without the mode converter are also shown. In both devices, the SHG occurs in the identically designed phase-matched straight waveguides. The key difference between two devices is the conversion of SHG signal from the TE_20_ mode to the TE_00_ mode by the mode converter. This conversion impacts the efficiency of light collection by the output lensed fiber, as light closer to the TE_00_ mode achieves better coupling. The results in Figure 4(b) show that the measured off-chip SHG power is approximately ∼152 nW for the reference waveguide and ∼335 nW for the waveguide with the inverse-designed mode converter, indicating an improvement in collection efficiency of about 2.20 times, as shown in Figure 3(b). While the modest improvement in the measured SHG power potentially results from unintended mode conversion within the waveguide, the inverse-designed device achieves a meaningful performance enhancement despite a 32.4 % decrease in transmission. Additionally, the differences in SHG peak wavelength between two devices, as well as the presence of additional smaller peaks and dips, are primarily attributed to fabrication-induced variations in waveguide geometry, such as thickness variation [47]. It is also important to note that the coupling efficiency is maximized during alignment, which introduces an empirical uncertainty of approximately ±5 % due to potential slight misalignment.

Next, to calculate the SHG efficiency, the coupling losses at the input and output facets are determined by comparing the transmissions of two waveguides: one with a tapered input and a straight output, and another with both straight input and output. The coupling efficiencies at both facets are measured at 775 nm and 1,550 nm. From these measurements, the coupling losses for both the tapered and straight facets are calculated. Considering the measured coupling loss of −10.41 dB and −7.12 dB for 1550-nm input facet and 775-nm output facet, respectively, the on-chip SHG conversion efficiency of the device including the mode converter is calculated to be approximately 6.66 % W^−1^ cm^−2^. The theoretical prediction for SHG efficiency is calculated as 63.3 % W^−1^ cm^−2^, based on the overlap integral of the interacting modes [48]. Since this calculation assumes ideal condition and excludes minor variations in waveguide dimensions and surface roughness attributed from the fabrication imperfections, the theoretical prediction aligns closely with the measured value. By improving experimental parameters such as transmission, coupling efficiency, and waveguide loss, we expect to achieve even higher efficiency values. Figure 4(c) shows the variation of SHG power as a function of pump power in telecom. The dashed line represents a quadratic fit to the measured data, suggesting that the SHG power increases quadratically with the pump power. The quadratic coefficient is 5.71 × 10^−5^ W^−1^, and the *R*-squared value is 0.986, confirming the quadratic relationship between SHG power and pump power.

### 3.2 Measurement of spontaneous parametric down conversion

**Figure 5:**
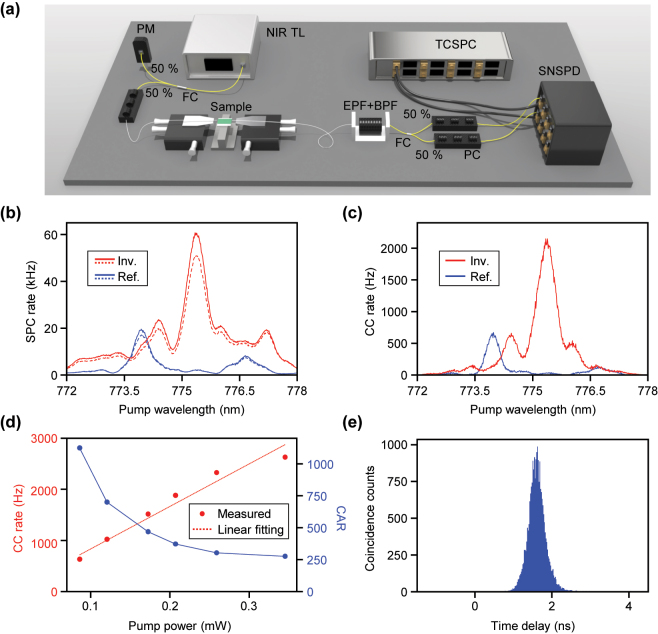
Experimental results of measuring SPDC. (a) Schematic illustration of experimental setup for measuring SPDC. TL, tunable laser; FC, fiber coupler; EPF, edge pass filter; BPF, band pass filter; PC, polarization controller; SNSPD, superconducting nanowire single photon detector; TCSPC, time-correlated single photon counter. (b) Measured single photon count (SPC) rate with respect to the pump wavelength. Solid and dotted lines indicate two different SNSPD channels. (c) Measured coincidence count (CC) rate with respect to the pump wavelength. (d) Measured coincidence count rate and coincidence-to-accidental ratio (CAR) with respect to the pump power. The dotted red line indicates a linear fitting of the CC rate. (e) Histogram of the coincidence counts with respect to the time delay between two SNSPD channels.

Next, we perform the experiment to measure the SPDC. The detailed experimental setup is schematically depicted in Figure 5(a). Light from a tunable near-infrared laser (New focus, TLB-6712) is first injected into a variational optical attenuator (VOA) and fiber polarization controller. The light is then split by a 50:50 fiber coupler, with one port used for power monitoring and another port directed to the sample. A 775 nm lensed fiber is used to couple the light into the sample. On-chip, the 775-nm light is mostly coupled to a straight waveguide with TE_00_ fundamental mode and tapered waveguide structures to ensure that only the fundamental modes propagate. Then, the TE_00_ modes are converted to TE_20_ modes by the freeform mode converter to achieve phase matching with the 1,550 nm TE_00_ mode. Under this condition, two 1550-nm photons are generated by SPDC from vacuum noise and are coupled out through the output lensed fiber, optimized for operation at 1,550 nm. Subsequently, three cascaded edge-pass filters are used to eliminate the 775 nm pump light, followed by a 1,550 nm center band pass filter with a 12 nm bandwidth to remove photons from other noise sources. The two photons are probabilistically separated by a 50:50 fiber coupler. Fiber polarization controllers are utilized to optimize detection efficiency for superconducting nanowire single photon detector (SNSPD). Finally, the photon arrival signals generated by the SNSPDs are recorded by a time-correlated single-photon counter (TCSPC).

Using the experimental setup illustrated in Figure 5(a), the spectra of the single-photon count rate and coincidence count rate are measured. The corresponding results are presented in Figure 5(b) and (c), respectively. For each wavelength, the exposure time is set to 1 s, and the coincidence window is set to 2 ns. For comparison, measurement results for the reference waveguide device without the mode converter are also provided. During the alignment process, the lensed fiber is intentionally misaligned to excite higher-order modes in the pump light, ensuring that the reference device operates under its optimal performance conditions, maximizing single and coincidence counts. Furthermore, unavoidable nanometer-scale sidewall roughness in the fabricated waveguide induces the excitation of higher-order modes along the waveguide length, which marginally contributes to the photon-pair generation rate. Compared to the reference waveguide, the single and coincidence count rates for the device with the mode converter are approximately ∼3.05 and ∼3.21 times higher, respectively. This shows that the pump light in the 775 nm band with TE_00_ mode is effectively converted to the TE_20_ mode by the mode converter. Additionally, it can be observed that the SHG spectra and the single/coincidence counts are nearly identical across the measured wavelength range. The SHG and SPDC peak wavelengths are measured to be 774.090 nm and 773.985 nm, respectively, for the reference waveguide, and 775.340 nm and 775.343 nm, respectively, for the waveguide with the mode converter. These results demonstrate a strong agreement between the them.

Figure 5(d) presents the measured coincidence count rate as a function of the pump power for our inversely designed mode converter. The VOA is adjusted to attenuate the 20 mW laser power to 345 μW at the fiber port before being coupled to the lensed fiber. It is observed that as the pump power increases, the coincidence count rate also increases. Unlike SHG, SPDC is a process in which a single pump photon is split into two photons (signal and idler) within a nonlinear medium. The rate of this process is directly proportional to the number of pump photons available, resulting in a linear dependence on pump power. In Figure 5(d), a linear fit of the measured data is represented by the red dotted line. The linear coefficient is calculated to be 8.321 kHz/mW, and the *R*-squared value is approximately 0.956, indicating a strong linear relationship. Accounting for fiber-to-chip coupling losses, an on-chip photon pair generation rate of 5.173 MHz/mW is achievable. Moreover, considering the detection efficiency of SNSPD (∼80 %), the actual on-chip generation rate is expected to be even higher. Similarly, the coincidence to accidental ratio (CAR) is found to be inversely proportional to increasing pump power, with the highest value of approximately 1,123 observed at pump power of 86.3 μW. Even at pump power of 345 μW, where the photon pair generation rate exceeds 2 kHz, the CAR remains at a relatively high value of 275.6. Figure 5(e) displays the histogram of the difference between the arrival times of each photon in channels 1 and 2. With an exposure time of 10 s, the time delay is observed to fall within a narrow 2 ns interval. This narrow peak in the histogram further validates the temporal correlation between the photon pairs generated by our freeform mode converter.

## 4 Discussion and conclusions

In this work, we propose an inverse design method tailored to address the severe fabrication constraints of the nonlinear TFLN platform, including challenges such as slanted sidewalls and a large minimum gap size exceeding half the wavelength. As a quantitative comparison, our design incorporates minimum width constraints in units of wavelength that are 2.3 times greater and minimum height sizes that are 4 times greater than those of recent work [40], highlighting the substantial restrictions imposed by fabrication constraints in our study. These unique challenges necessitated careful adaptation and represent a significant contribution to the field of nanophotonic device design under stringent fabrication limitations. Using this approach, we successfully demonstrate a freeform TFLN mode converter with a compact footprint, low loss, high modal purity, and strong agreement between simulation and experimental results. The proposed inverse-design methodology is not limited to the spatial mode converter but applicable to various design problems of advanced TFLN devices on both linear and nonlinear platforms, such as logic gates [34], [49], cavity resonators [33], [50], [51], and wavelength demultiplexer [52], [53]. Additionally, reducing the etching depth through the use of periodic-poling can mitigate fabrication constraints [54], allowing our proposed method to enable the design of more efficient yet compact LN-based devices.

Compared to our previous work on shape-optimized mode converter [21], which was limited by larger footprints and suboptimal performance due to constraints in design space and assumptions of perpendicular sidewalls, our current inverse-designed freeform mode converter offers significant advantages. By employing the proposed inverse design framework, we have achieved a much smaller device footprint of 5.6 × 7.6 μm^2^ compared to the 15 μm length of the previous design. Despite the reduced footprint of our proposed device, the product of CC rate and CAR of ours is 7.11 × 10^5^, which is an improvement over the previous design’s 3.14 × 10^5^. Our framework overcomes the limitations of previous methods by effectively navigating complex fabrication constraints while improving device performances.

Although the photon pair generation rate of our mode converter is lower than periodically poled lithium niobate (PPLN) devices, it offers distinct advantages that make it a compelling alternative for specific applications. The proposed mode converter provides a broader design space, enabling higher-order mode engineering and more compact device footprints. Unlike PPLN devices, our approach does not rely on periodic poling, which is particularly challenging for *z*-cut TFLN platforms and requires a complex fabrication process. Moreover, the performance of PPLN devices is highly sensitive to fabrication imperfections, potentially limiting their scalability in densely integrated photonic circuits. In contrast, the mode converter-based approach demonstrated here offers a versatile and robust solution, particularly suited for applications demanding high integration density and simplified single-step fabrication.

The fabricated freeform TFLN mode converter in Figure 3(f) exhibits minor deviations from the designed structure in Figure 3(e), including small cuts on the top-right and bottom-right hole as well as slightly reduced hole size. Despite these slight discrepancies, the critical structural features essential for the device’s functionality-such as the overall geometry and alignment-remain preserved. Experimental validation confirms that these minor variations have a negligible impact on the device’s performance. While the fabricated freeform TFLN mode converter has demonstrated effectiveness through SHG and SPDC measurements, there are several potential improvements that can further improve its performance. The low fiber-to-chip coupling efficiency is one of the critical factors that need improvements. Optimizing the taper design at both the input and output interfaces can significantly improve the mode matching between the fiber and the waveguide. Fine-tuning the taper shape to enable more adiabatic mode conversion can minimize the insertion loss and improve the overall coupling efficiency [55], [56], [57]. Another factor that affects the device performance is the waveguide loss due to scattering, which is mainly caused by fabrication defects such as surface roughness introduced during the etching process. The scattering loss can be mitigated by refining the fabrication process to achieve smoother sidewalls and reduce surface irregularities [58]. In addition, the variation of the TFLN thickness can affect the nonlinear process, as the non-uniform thickness can cause the phase mismatch and reduce nonlinear interaction efficiency [47], [59]. Moreover, reducing the smallest feature size achievable in the device can enhance performance. This increased design freedom allows for more intricate and optimized structure, resulting in more compact and high-performance devices.

In conclusion, we introduced the freeform inverse-designed devices into TFLN quantum nonlinear photonics and demonstrated the potential in experiments for the first time. Our inverse design framework effectively addresses critical fabrication constraints in TFLN-based integrated photonic devices, enabling the design of devices with enhanced performances and functionalities. The successful experimental implementation of the mode converter validates the effectiveness of the framework and opens up new opportunities for the exploitation of various modes in TFLN waveguides. We anticipate that this framework can be a valuable tool for advancing the development of highly efficient and custom photonic devices. Extending this approach to similar material platforms can significantly contribute to high-performance applications in nonlinear optics and quantum photonics.

## References

[1] Dutt A., Mohanty A., Gaeta A. L., Lipson M. (2024). Nonlinear and quantum photonics using integrated optical materials. *Nat. Rev. Mater.*.

[2] Bao J. (2023). Very-large-scale integrated quantum graph photonics. *Nat. Photonics*.

[3] Pelucchi E. (2022). The potential and global outlook of integrated photonics for quantum technologies. *Nat. Rev. Phys.*.

[4] Wang J., Sciarrino F., Laing A., Thompson M. G. (2020). Integrated photonic quantum technologies. *Nat. Photonics*.

[5] Qi Y., Li Y. (2020). Integrated lithium niobate photonics. *Nanophotonics*.

[6] Zhu D. (2021). Integrated photonics on thin-film lithium niobate. *Adv. Opt. Photonics*.

[7] Saravi S., Pertsch T., Setzpfandt F. (2021). Lithium niobate on insulator: an emerging platform for integrated quantum photonics. *Adv. Opt. Mater.*.

[8] Vazimali M. G., Fathpour S. (2022). Applications of thin-film lithium niobate in nonlinear integrated photonics. *Adv. Photonics*.

[9] Boes A. (2023). Lithium niobate photonics: unlocking the electromagnetic spectrum. *Science*.

[10] Zhang M., Wang C., Kharel P., Zhu D., Lončar M. (2021). Integrated lithium niobate electro-optic modulators: when performance meets scalability. *Optica*.

[11] Luke K., Kharel P., Reimer C., He L., Loncar M., Zhang M. (2020). Wafer-scale low-loss lithium niobate photonic integrated circuits. *Opt. Express*.

[12] Wang C. (2018). Integrated lithium niobate electro-optic modulators operating at cmos-compatible voltages. *Nature*.

[13] Desiatov B., Shams-Ansari A., Zhang M., Wang C., Lončar M. (2019). Ultra-low-loss integrated visible photonics using thin-film lithium niobate. *Optica*.

[14] Xu M. (2020). High-performance coherent optical modulators based on thin-film lithium niobate platform. *Nat. Commun.*.

[15] Wang C., Zhang M., Yu M., Zhu R., Hu H., Loncar M. (2019). Monolithic lithium niobate photonic circuits for kerr frequency comb generation and modulation. *Nat. Commun.*.

[16] Zhang M. (2019). Broadband electro-optic frequency comb generation in a lithium niobate microring resonator. *Nature*.

[17] Chen P.-K., Briggs I., Hou S., Fan L. (2022). Ultra-broadband quadrature squeezing with thin-film lithium niobate nanophotonics. *Opt. Lett.*.

[18] Stokowski H. S. (2023). Integrated quantum optical phase sensor in thin film lithium niobate. *Nat. Commun.*.

[19] Lu J., Al Sayem A., Gong Z., Surya J. B., Zou C.-L., Tang H. X. (2021). Ultralow-threshold thin-film lithium niobate optical parametric oscillator. *Optica*.

[20] Zhao J., Ma C., Rüsing M., Mookherjea S. (2020). High quality entangled photon pair generation in periodically poled thin-film lithium niobate waveguides. *Phys. Rev. Lett.*.

[21] Kwon K. (2024). Photon-pair generation using inverse-designed thin-film lithium niobate mode converters. *APL Photonics*.

[22] Ledezma L., Sekine R., Guo Q., Nehra R., Jahani S., Marandi A. (2022). Intense optical parametric amplification in dispersion-engineered nanophotonic lithium niobate waveguides. *Optica*.

[23] Song Y., Hu Y., Zhu X., Yang K., Lončar M. (2024). Octave-spanning kerr soliton frequency combs in dispersion-and dissipation-engineered lithium niobate microresonators. *Light: Sci. Appl.*.

[24] Briggs I., Hou S., Cui C., Fan L. (2021). Simultaneous type-i and type-ii phase matching for second-order nonlinearity in integrated lithium niobate waveguide. *Opt. Express*.

[25] Chapman R. J., Häusler S., Finco G., Kaufmann F., Grange R. (2023). Quantum logical controlled-not gate in a lithium niobate-on-insulator photonic quantum walk. *Quantum Sci. Technol.*.

[26] Bendsoe M. P., Sigmund O. (2013). *Topology Optimization: Theory, Methods, and Applications*.

[27] Molesky S., Lin Z., Piggott A. Y., Jin W., Vucković J., Rodriguez A. W. (2018). Inverse design in nanophotonics. *Nat. Photonics*.

[28] Kang C., Park C., Lee M., Kang J., Jang M. S., Chung H. (2024). Large-scale photonic inverse design: computational challenges and breakthroughs. *Nanophotonics*.

[29] Li Z., Pestourie R., Park J.-S., Huang Y.-W., Johnson S. G., Capasso F. (2022). Inverse design enables large-scale high-performance meta-optics reshaping virtual reality. *Nat. Commun.*.

[30] Bae M., Jo J., Lee M., Kang J., Boriskina S. V., Chung H. (2023). Inverse design and optical vortex manipulation for thin-film absorption enhancement. *Nanophotonics*.

[31] Roberts G. (2023). 3d-patterned inverse-designed mid-infrared metaoptics. *Nat. Commun.*.

[32] Chung H., Miller O. D. (2020). Tunable metasurface inverse design for 80 % switching efficiencies and 144 angular deflection. *ACS Photonics*.

[33] Yang J., Guidry M. A., Lukin D. M., Yang K., Vučković J. (2023). Inverse-designed silicon carbide quantum and nonlinear photonics. *Light: Sci. Appl.*.

[34] He L. (2023). Super-compact universal quantum logic gates with inverse-designed elements. *Sci. Adv.*.

[35] Lee S. (2024). Inverse design of color routers in cmos image sensors: toward minimizing interpixel crosstalk. *Nanophotonics*.

[36] Jo J. (2024). Inverse designed ws2 planar chiral metasurface with geometric phase. *J. Opt.*.

[37] Kim C. (2024). Freeform metasurface color router for deep submicron pixel image sensors. *Sci. Adv.*.

[38] Choi T. (2024). Multiwavelength achromatic deflector in the visible using a single-layer freeform metasurface. *Nano Lett.*.

[39] Miller O. D. (2012). *Photonic Design: From Fundamental Solar Cell Physics to Computational Inverse Design*.

[40] Shang C. (2023). Inverse-designed lithium niobate nanophotonics. *ACS Photonics*.

[41] Zhao X., Shi Z., Chen Q. (2020). Inverse design of an indoor environment using a filter-based topology method with experimental verification. *Indoor air*.

[42] Hammond A. M., Oskooi A., Chen M., Lin Z., Johnson S. G., Ralph S. E. (2022). High-performance hybrid time/frequency-domain topology optimization for large-scale photonics inverse design. *Opt. Express*.

[43] Chen M. (2024). Validation and characterization of algorithms and software for photonics inverse design. *JOSA B*.

[44] Hägg L., Wadbro E. (2018). On minimum length scale control in density based topology optimization. *Struct. Multidiscip. Optim.*.

[45] Oskooi A. F., Roundy D., Ibanescu M., Bermel P., Joannopoulos J. D., Johnson S. G. (2010). Meep: a flexible free-software package for electromagnetic simulations by the fdtd method. *Comput. Phys. Commun.*.

[46] Christiansen R. E., Sigmund O. (2021). Inverse design in photonics by topology optimization: tutorial. *JOSA B*.

[47] Chen P.-K., Briggs I., Cui C., Zhang L., Shah M., Fan L. (2024). Adapted poling to break the nonlinear efficiency limit in nanophotonic lithium niobate waveguides. *Nat. Nanotechnol.*.

[48] Jankowski M., Mishra J., Fejer M. (2021). Dispersion-engineered nanophotonics: a flexible tool for nonclassical light. *J. Phys.: Photonics*.

[49] Wang H., Xu H., Huang H., Zhou N., Zhang H., Li J. Ultra-broadband and ultra-compact chip-integrated logic gates based on an inverse design method. *Opt Laser. Technol.*.

[50] Ahn G. H. (2022). Photonic inverse design of on-chip microresonators. *ACS Photonics*.

[51] Bi T. (2024). Inverse designed silicon nitride photonic linear microresonators. *CLEO: Science and Innovations*.

[52] Piggott A. Y., Lu J., Lagoudakis K. G., Petykiewicz J., Babinec T. M., Vučković J. (2015). Inverse design and demonstration of a compact and broadband on-chip wavelength demultiplexer. *Nat. Photonics*.

[53] Guo X., Zou C.-L., Schuck C., Jung H., Cheng R., Tang H. X. (2017). Parametric down-conversion photon-pair source on a nanophotonic chip. *Light: Sci. Appl.*.

[54] Yang F., Lu J., Shen M., Yang G., Tang H. X. (2024). Symmetric second-harmonic generation in sub-wavelength periodically poled thin film lithium niobate. *Optica*.

[55] He L., Zhang M., Shams-Ansari A., Zhu R., Wang C., Marko L. (2019). Low-loss fiber-to-chip interface for lithium niobate photonic integrated circuits. *Opt. Lett.*.

[56] Liu X. (2022). Ultra-broadband and low-loss edge coupler for highly efficient second harmonic generation in thin-film lithium niobate. *Adv. Photonics Nexus*.

[57] Hu C. (2021). High-efficient coupler for thin-film lithium niobate waveguide devices. *Opt. Express*.

[58] Zhu X. (2024). Twenty-nine million intrinsic q-factor monolithic microresonators on thin-film lithium niobate. *Photonics Res.*.

[59] Zhao J. (2023). Unveiling the origins of quasi-phase matching spectral imperfections in thin-film lithium niobate frequency doublers. *APL Photonics*.

